# Optimization of New Cropland Allocation to Enhance Stable Utilization Potential: A Case Study of Guangdong Province, China

**DOI:** 10.3390/foods15111845

**Published:** 2026-05-23

**Authors:** Lesong Zhao, Ziyuan Qiao, Guangsheng Liu, Hongmei Wang

**Affiliations:** 1 School of Public Administration, South China Agricultural University, Guangzhou 510642, China; 2 Guangdong Province Key Laboratory for Agricultural Resources Utilization, College of Natural Resources and Environment, South China Agricultural University, Guangzhou 510642, China; 3 Key Laboratory of Natural Resources Monitoring in Tropical and Subtropical Area of South China, Ministry of Natural Resources, Guangzhou 510700, China

**Keywords:** new cropland, stable utilization potential, optimized allocation, scenario comparison, Guangdong Province

## Abstract

The optimization of new cropland allocation is crucial for promoting the efficient use of cropland resources and safeguarding food security. However, existing studies primarily take suitability as the optimization objective and lack the consideration of stable utilization potential, which may lead to subsequent unstable use. To address this gap, this study quantified the stable utilization potential of new cropland using a machine learning model and integrated it with the ant colony optimization (ACO) model to develop a spatial allocation framework. This framework was validated in Guangdong Province, China, a region characterized by diverse resource endowments and pronounced regional heterogeneity. The results indicated that, under the specified objective-weighting scheme and compared with the business-as-usual (BAU) scenario, the optimized scenario achieved regional cropland quantity balance. It also increased the average stable utilization potential of new cropland and overall utility by 34.84% and 12.74%, respectively, while reducing the mean cost and mean ecological benefit loss per unit area by 10.22% and 41.36%, respectively. Overall, under the specified constraints, the proposed framework offers a promising approach for new cropland planning and provides a basis for governments and land management authorities to improve future allocation practice.

## 1. Introduction

Cropland is not only fundamental to food security, but also performs multiple functions related to the supply of important agricultural products, the maintenance of ecological functions, and the safeguarding of social stability [1,2,3]. As such, it constitutes an essential foundation for achieving the United Nations Sustainable Development Goal of “Zero Hunger” and serves as a vital basis for human survival and socioeconomic development [4,5]. With continued global population growth and shifts in consumption patterns, global food demand is projected to increase substantially by 2050, and the Food and Agriculture Organization of the United Nations (FAO) estimates that global cropland area will need to expand by approximately 69 million ha relative to 2005 to meet this rising demand [6,7]. This trend may stimulate both cropland intensification and expansion, thereby generating a considerable amount of new cropland [2,8].

New cropland represents an important component of cropland resources and a critical basis for cropland protection [9]. Compared with increasing yield per unit area through agricultural technological advancement, expanding the cropland area through the creation of new cropland is often regarded as a common strategy for alleviating regional cropland constraints and enhancing grain production [8,10]. In developing countries such as China, new cropland is also important for maintaining the balance of total cropland area and compensating for losses caused by rapid urbanization and industrialization [11], and more than 2.63 million ha of cropland were supplemented annually on average in China during 2000–2020, providing important support for safeguarding the cropland quantity red line [12]. In addition, new cropland creation can be integrated with the optimization of the cropland spatial layout, thereby promoting the concentration of cropland in areas more suitable for cultivation and improving the overall quality and productive capacity of regional cropland [13,14]. Therefore, new cropland plays a crucial role in maintaining regional cropland quantity balance, optimizing cropland spatial patterns, and strengthening food production capacity.

Over the past three centuries, global cropland area has expanded nearly fivefold, generating a substantial amount of new cropland [15] and contributing positively to food provision and social stability. However, existing studies have shown that a considerable proportion of new cropland has been converted from grassland, forest land, and other land types characterized by relatively poor water and heat conditions, unfavorable terrain, and low soil quality. As a result, such land generally remains inferior in overall quality to base cropland, even after the provision of field roads and irrigation infrastructure [9,10]. In China, the creation of new cropland has largely occurred in response to the requisition–compensation balance policy, with the primary aim of offsetting historical losses in cropland productivity [11,16]. Cropland reclamation has therefore often been dominated by government-led administrative actions, and the site selection of new cropland has not fully incorporated farmers’ preferences [11,17]. Constrained by costs and resource endowments, a considerable proportion of new cropland has been located in marginal areas such as hilly and mountainous regions. In these areas, relatively low economic returns and ecological disturbance jointly contribute to declining productivity, conversion to other land uses, or abandonment, thereby undermining sustained cultivation [9,18,19,20]. Moreover, some scholars have evaluated the long-term utilization of new cropland and found that more than 45% of historical new cropland experienced abandonment at least once during the study period [9,19]. This not only results in a substantial waste of resource inputs devoted to new cropland consolidation, but also undermines the effectiveness of cropland protection policies and poses a significant threat to regional food security. To address these issues, China has reformed its requisition–compensation balance policy and further emphasized the critical role of stable utilization in future cropland supplementation. Therefore, improving the stable utilization of new cropland is of great significance for safeguarding regional food security and promoting the high-quality use of land resources.

Existing studies have paid relatively limited attention to the stable utilization of new cropland, with research mainly focusing on two aspects. The first concerns the assessment of the stable utilization of historical new cropland. Most studies have relied on continuous land use data to evaluate new cropland utilization from the perspective of abandonment over extended periods, measuring long-term utilization stability in terms of the duration of stable use as well as the frequency and timing of conversion to other land uses [9,10,18,19]. Spatial overlay analysis has also been used to calculate cropland supplementation and abandonment rates and to characterize their spatial relationships [12], while the utilization level of new cropland has also been examined from the perspective of sustainable use [21]. The second aspect focuses on identifying the key factors underlying differences in stable utilization. Econometric methods such as logistic regression and nonlinear methods such as boosted regression trees have been used to reveal the effects of natural and locational conditions, neighborhood environments, and human activities on stable utilization differences [12,19,22]. Owing to differences in study areas and methods, the key influencing factors identified also vary. Overall, existing studies have largely addressed whether historical new cropland has been stably utilized and why instability occurs. However, these studies remain mainly at the level of historical assessment and mechanism explanation, and their findings have not yet been effectively translated into a basis for the future optimal allocation of new cropland.

The optimal allocation of new cropland is critical to enhancing its stable utilization potential, as it determines the regional composition and spatial configuration of future cropland expansion and thereby shapes long-term stability and overall utility. However, existing studies have largely relied on suitability assessment or land simulation approaches, focusing primarily on objectives such as maximizing agricultural production potential or suitability [23,24], reducing costs [17] and minimizing ecological losses [25,26]. Stable utilization potential has not yet been incorporated as a quantifiable and comparable core objective, and little attention has been paid to integrating it into a unified framework that also considers cropland development costs and expansion-scale constraints. This omission may result in new cropland that remains difficult to use stably after development, thereby undermining its long-term sustainable utilization.

In summary, to address the limitations of existing studies, this study takes Guangdong Province, China, as the study area and develops two scenarios: a business-as-usual (BAU) scenario and a stable utilization potential enhancement (SUPE) scenario. First, the allocation pattern of new cropland under the BAU scenario is identified. On this basis, an optimized allocation scheme for new cropland under the SUPE scenario is developed, with the enhancement of stable utilization potential as the central objective. The optimization effects are then evaluated through comparison with the BAU scenario. This paper aims to answer the following three questions: (1) How does the stable utilization potential of future new cropland vary across space? (2) How can future new cropland be optimally allocated to enhance stable utilization potential under multiple objectives and constraints? (3) Compared with the BAU scenario, can the optimized scenario achieve better allocation outcomes?

## 2. Materials and Methods

### 2.1. Study Area

**Figure 1:**
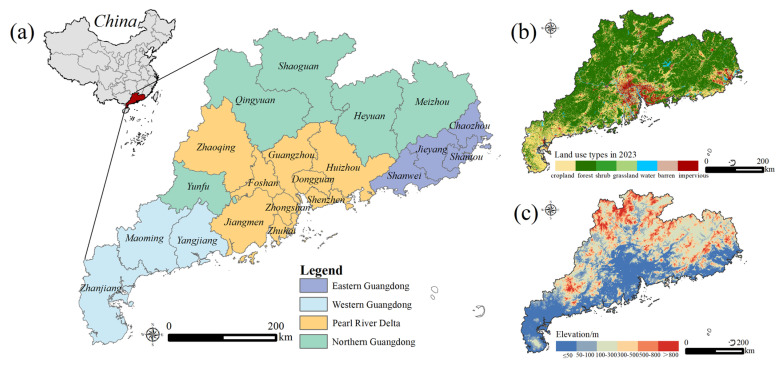
Location of the study area: (**a**) location of Guangdong in China; (**b**) land use types of Guangdong; and (**c**) elevation of Guangdong.

Guangdong Province is located in southern mainland China, extending from 109°45′ to 117°20′ E and from 20°09′ to 25°31′ N, with a total area of approximately 179,800 km^2^. According to differences in geographical location and economic development, the province can be divided into four subregions: eastern Guangdong, western Guangdong, northern Guangdong, and the Pearl River Delta (Figure 1). Guangdong lies within the East Asian monsoon region and is characterized by favorable light and heat conditions, abundant precipitation, a marked synchrony between rainfall and heat, and a dense river network, providing favorable conditions for agricultural production. It also exhibits diverse landforms, including plains, tablelands, hills, and mountains, with higher elevations in the north and lower elevations in the south. This complex topography has given rise to pronounced spatial heterogeneity in natural conditions, development intensity, and the potential for new cropland, making Guangdong a representative case for the spatial optimization of new cropland allocation.

In addition, Guangdong had a permanent resident population of approximately 127 million by the end of 2023, and its GDP reached RMB 13.57 trillion in 2023, both of which have ranked first among provincial-level regions in China for many consecutive years. A relatively limited land area therefore supports an exceptionally large population and economic scale, resulting in strong demand for construction land and considerable pressure on cropland protection and food security. As a consequence, human–land conflicts are particularly pronounced, and optimizing future new cropland allocation is more complex and urgent. Therefore, selecting Guangdong Province as the study area helps capture the differentiated demand for new cropland optimization under regional heterogeneity and provides a reference for regions facing similar human–land conflicts and resource constraints.

### 2.2. Data Sources

The data used in this study mainly comprised two types: vector and raster data, and their sources are summarized in Table 1. The vector data mainly included: (1) Administrative boundary data for Guangdong Province, which were primarily used for regional division and statistical analysis. (2) The Third National Land Survey data of Guangdong Province, which were mainly used to extract layers such as ditches, roads, and the spatial extent of urban, rural, industrial, and mining land areas. (3) Ecological redline data, which were mainly used as ecological constraint conditions. The raster data mainly included the following categories: (1) Digital Elevation Model (DEM) data, with a spatial resolution of 30 m, which were mainly used to quantify elevation, slope, and terrain relief. (2) Soil data obtained from the Soil SubCenter of the National Earth System Science Data Center, National Science and Technology Infrastructure of China, with a spatial resolution of 1 km, which were mainly used to derive soil-related indicators. (3) Land use data derived from the 30 m resolution CLCD produced by Wuhan University and a land use dataset produced by the Chinese Academy of Sciences (CNLUCC). Among these, the CLCD was developed from Landsat imagery on the Google Earth Engine (GEE) platform and provides annual land cover products for China from 1985 onward, with an overall accuracy of 79.31% [27]. It was mainly used for identifying the candidate units and quantifying the driving factors used in the machine learning model. The other dataset was obtained from the Resource and Environment Science and Data Center of the Chinese Academy of Sciences and was mainly used to extract historical rural settlements and urban land. (4) Night-time light (NTL) data, with a spatial resolution of 1 km [28], were mainly used for driving factor quantification and land use simulation. (5) Precipitation and temperature data obtained from the National Tibetan Plateau Data Center, with a spatial resolution of 1 km [29,30,31,32,33], were mainly used for driving factor quantification and land use simulation. (6) Population data obtained from the WorldPop dataset, with a spatial resolution of 100 m, were mainly used for factor quantification and land use simulation.

### 2.3. Methods

#### 2.3.1. Research Framework

**Figure 2:**
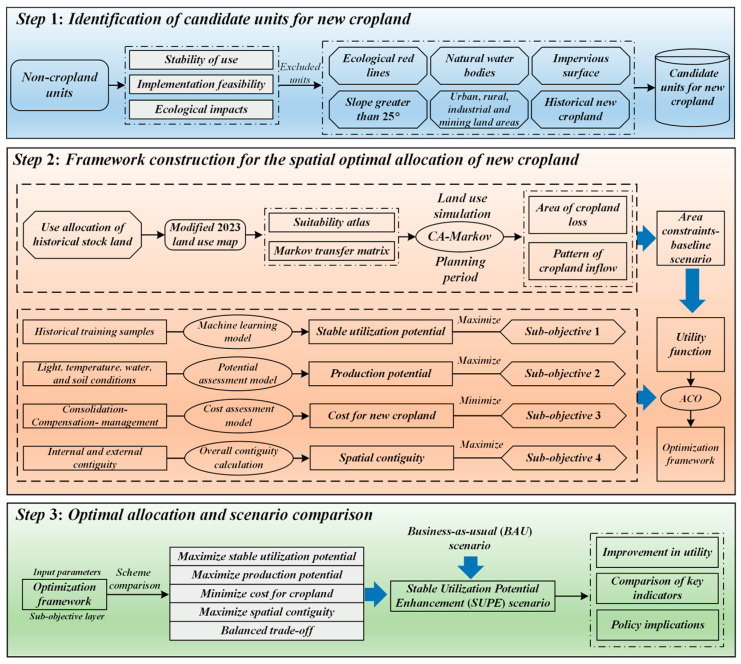
Research framework.

The optimization of new cropland allocation mainly involves three steps (Figure 2). (1) Identification of candidate units for new cropland. In this study, the research objects are defined as candidate units for new cropland, referring to land units that can be converted into new cropland in the future through appropriate land consolidation measures under given constraints. To ensure the stability and feasibility of future new cropland while minimizing potential adverse ecological impacts, units located within the ecological redline, with slopes greater than 25°, within urban, rural, industrial, and mining land areas, or with terminal land use types of natural water bodies or impervious surfaces were excluded. In addition, land units that had already been identified as historical new cropland during the study period were also excluded from the candidate pool. (2) Construction of the spatial optimization framework for new cropland. First, a land use simulation model was used to determine cropland loss and the spatial pattern of new cropland under the business-as-usual (BAU) scenario for 2035, providing the basis for the future area constraint and the baseline scenario. Second, a utility function was constructed by integrating four sub-objectives, namely maximizing stable utilization potential, maximizing production potential, minimizing cost, and maximizing spatial contiguity. Third, by coupling the sub-objective functions with the ACO algorithm, a spatial optimization framework for new cropland was established to support its future allocation. (3) Optimal allocation and scenario comparison. Based on the proposed framework, sensitivity analysis was conducted to determine the parameter settings for the stable utilization potential enhancement (SUPE) scenario and thereby achieve the spatial optimization of new cropland. This scenario was then compared with the baseline scenario to quantify improvements in overall utility and key indicators.

#### 2.3.2. Utility Function Construction for New Cropland Allocation Optimization

The optimization of land use allocation is usually a type of problem that needs to consider multiple objectives [34,35]. Accordingly, the optimization of new cropland allocation is inherently a multi-objective spatial optimization process conducted under multiple constraints, rather than a task driven by a single objective. More specifically, in addition to stable utilization potential, this study considers other key objectives, including production potential, cost for new cropland, and spatial contiguity. Production potential reflects the capacity of newly created cropland to sustain agricultural output and provides an essential basis for ensuring cropland quality, crop production capacity, and regional food security [36]. Cost for new cropland represents the economic feasibility of land consolidation, engineering construction, and subsequent use, and thus constitutes an important constraint on the practical implementation of optimized allocation [17]. Spatial contiguity is closely related to the conditions for large-scale farming of future cropland and further influences agricultural production efficiency and long-term land use stability [37,38]. Accordingly, this study develops a multi-objective utility function centered on stable utilization potential while also incorporating production potential, the cost for new cropland, and spatial contiguity, so as to achieve a coordinated optimization of future new cropland in terms of stable use, quality, and spatial configuration. The objective function for future new cropland allocation is defined as follows:

(1) $$U_{c}=w_{1}K_{1}+w_{2}K_{2}-w_{3}K_{3}+w_{4}K_{4}$$

where *U_c_* represents the utility function for future new cropland allocation; *w_i_* represents the weight assigned to each sub-objective (*i* = 1, 2, 3, 4); and *K_i_* represents each sub-objective (*i* = 1, 2, 3, 4).

(1) Maximize Stable Utilization Potential

Maximizing stable utilization potential refers to an allocation principle that gives priority to candidate units with a greater likelihood of sustaining cropland use in the long term, so as to enhance the overall stable-use capacity of future new cropland under given planning constraints. To estimate the stable utilization potential of future new cropland, a prediction model was developed based on eXtreme Gradient Boosting (XGBoost). XGBoost is an efficient tree-based learning algorithm within the Gradient Boosting framework and is widely regarded as a representative supervised learning method. Its central idea is to construct an additive model in a stage-wise manner by sequentially integrating multiple decision trees. Each new tree is fitted to further reduce the loss function, thereby combining weak learners into a more powerful predictive model [39]. By incorporating a regularization term into the objective function and penalizing tree complexity, XGBoost can effectively control overfitting and improve model generalization. This enhances predictive accuracy while avoiding unnecessarily complex model structures. In addition, because it does not require predictors to be free of collinearity, the method is generally insensitive to multicollinearity at the prediction stage [40]. Owing to these advantages, XGBoost has been widely applied in both classification and regression tasks [41,42,43,44].

To predict stable utilization potential, historical new cropland was first identified from 2001 to 2020 using the CLCD and each unit was assigned a corresponding temporal label. A three-year sliding window approach was then employed to determine the utilization status of new cropland [9,19]. On this basis, samples were constructed according to the conversion characteristics of historical new cropland and used to predict the stable utilization potential of future new cropland. Positive samples referred to units that remained under cropland use continuously for at least six years after their formation, corresponding to two consecutive three-year windows, whereas negative samples referred to units that underwent land use conversion after cropland formation and remained continuously under non-cropland use for at least six years. Subsequently, predictor variables were compiled from four dimensions, namely natural endowment, agricultural production and management, socioeconomic conditions, and neighborhood land use structure, and the specific variables together with their feature importance rankings are reported in Supplementary Table S1. These variables were organized into a feature matrix for model training under a five-fold cross-validation framework. Bayesian optimization was used to tune the key hyperparameters of the XGBoost model, and the optimal parameter combination was determined according to validation performance. Model performance was evaluated using overall accuracy, the area under the receiver operating characteristic curve (AUC), and related metrics [45]. The trained model was applied to candidate units for new cropland to generate the predicted probability that each unit would remain in a stable cropland state [44]. This probability was subsequently incorporated into the objective function for new cropland allocation as an indicator of stable utilization potential.

(2) Maximize Production Potential

Maximizing production potential refers to the preferential allocation of candidate units for new cropland based on their production potential, under constraints such as the required scale of new cropland during the planning period, to enhance the overall productive capacity of new cropland. Given the wide range of source land use types and broad spatial distribution of candidate units for new cropland [17,46,47], this objective helps prioritize units with superior resource endowments and higher potential productivity, thereby improving the overall output capacity and quality of future new cropland. In this study, the production potential of candidate units for new cropland was assessed using a stepwise correction approach. This approach is based on the light–temperature (climatic) productivity potential index and is subsequently adjusted through a series of corrections according to topographic characteristics, soil properties, and irrigation conditions, ultimately generating a cropland productivity score to represent production potential. This method has been widely applied in the assessment of cropland production potential [14,17,48,49,50]. Specifically, topographic characteristics include terrain slope and elevation [51,52]; soil properties include soil depth, soil texture, soil organic carbon content, and soil pH [14,50]; and irrigation conditions are measured by distance to water sources [53]. The production potential is calculated as follows:

(2) $$NACP_{i}={LP}_{i}\times {Ter}_{i}\times {Soi}_{i}\times {Irr}_{i}$$

(3) $${Ter}_{i}/{Soi}_{i}/{Irr}_{i}=\sum_{j=1}^{n}w_{j}\times I_{ij}/100$$

where *NACP_i_* represents the production potential of the *i*th unit; *Ter_i_*, *Soi_i_*, and *Irr_i_* represent the topographic characteristic index, soil property index, and irrigation condition index of the *i*th unit, respectively; *w_j_* represents the weight of the *j*th indicator; *I_ij_* represents the score of the *j*th indicator for the *i*th unit; and *n* represents the number of indicators.

(3) Minimize Cost

Cost is an important factor shaping the allocation of future new cropland. Under conditions of limited resources, excessively high investment may undermine economic viability and even prevent new cropland from meeting required quality standards, thereby affecting its stable utilization and weakening the effectiveness of cropland protection policies [17,54]. In this study, cost for new cropland refers to the expenditures incurred in converting candidate units into new cropland through engineering improvement or technical measures and in supporting their subsequent use and management, including consolidation cost, ecological compensation cost, and post-management and protection cost (PMPC). Consolidation cost refers to the engineering inputs required to transform candidate units into new cropland, including both direct conversion cost and the cost of improving agricultural production conditions. Ecological compensation cost refers to the expenditure associated with ecological function loss and corresponding compensation arising from the occupation of ecological land or changes in its original land use state during new cropland allocation. The estimation of consolidation cost and ecological compensation cost followed the method reported in the literature [17], with adjustments made to reflect the specific conditions of this study.

After the completion of new cropland projects, the absence of effective protection and management measures may expose new cropland to substantial risks of conversion away from grain production, abandonment, or conversion to construction land [19,55,56]. This not only wastes the resources invested in the earlier stages, but also undermines the expected benefits of cropland protection policies. It is therefore necessary to incorporate PMPC into the evaluation framework. In this study, PMPC was estimated using three indicators, namely cross-village coordination cost (CVCC), management accessibility cost (MAC), and neighborhood management and protection cost (NMPC). Among them, CVCC refers to the additional organizational and supervisory inputs required in the post-management stage when contiguous cropland patches extend across multiple administrative villages. It was measured by the number of administrative villages spanned by each contiguous cropland patch. The formulas for calculating CVCC are as follows:

(4) $${MC}_{i}=\sum_{j=1}^{m}z_{ij}$$

(5) $$z_{ij}=\left\{\begin{array}{cc} 1, & A_{ij}>0\\ 0, & A_{ij}=0 \end{array}\right.$$

where *MC_i_* represents the CVCC of the *i*th candidate unit; *z_ij_* represents a binary variable indicating whether candidate unit *i* overlaps with administrative village *j*; *A_ij_* represents the area of candidate unit *i* covered by administrative village *j*; and *m* represents the total number of administrative villages.

MAC was used to characterize the level of supervisory input associated with the spatial proximity of candidate units to the nearest management site. In this study, it was quantified by the distance from each candidate unit to the nearest village or town management site, with a greater distance indicating higher time and labor inputs for supervision, patrol, and routine management [17]. The formula for calculating MAC is as follows:

(6) $${SA}_{i}\left(d_{r}\right)=S_{k}^{r}\;\;\;d_{r}\in D_{k}^{r}$$

where *SA_i_*(*d_r_*) represents the MAC of the *i*th candidate unit; $D_{k}^{r}$ represents the distance class interval of the *k*th management and protection node; $S_{k}^{r}$ represents the score corresponding to that interval; and $d_{r}$ represents the distance from candidate unit *i* to the nearest management and protection node.

NMPC was used to capture the influence of the continuity of surrounding agricultural land and the convenience of management organization on management cost. More dispersed spatial patterns and weaker contiguity, as measured by the proportion of cropland and candidate units within a given neighborhood range, generally increase the organizational difficulty of patrol, maintenance, and operational scheduling; reduce management efficiency; raise post-management inputs; and thereby increase the risk of unstable utilization of new cropland [57]. The formula for calculating NMPC is as follows:

(7) $${NMC}_{i}\left(c\right)=S_{k}^{c}\;\;c\in Z_{k}$$

where *Z_k_* represents the *k*th proportion class interval; $S_{k}^{c}$ represents the score corresponding to the *k*th proportion class interval; and *c* represents the proportion of unit *i*.

To eliminate differences in units and magnitude among different cost components, the constructed cost evaluation indicators were normalized, and a weighted linear combination model was then used to calculate the total cost. The total cost is calculated as follows:

(8) $${TNAC}_{i}=\sum_{l=1}^{m}P_{j}\times S_{ij}$$

where *TNAC_i_* represents the total cost of the *i*th candidate unit; *P_j_* represents the weight of the *j*th cost indicator; *S_ij_* represents the normalized value of the *j*th cost indicator for the *i*th candidate unit; and *m* represents the number of cost indicators.

(4) Maximize Spatial Contiguity

Cropland spatial contiguity can substantially affect the stable utilization of cropland by shaping agricultural production and management conditions [19,58]. In this study, the spatial contiguity objective incorporates both neighborhood connectivity among new cropland units and their spatial adjacency to existing cropland, thereby encouraging a more clustered spatial pattern and stronger integration with the existing cropland system. Overall spatial contiguity was quantified at the raster level based on shared edges using a four-neighbor adjacency rule, in terms of two dimensions: the internal aggregation of new cropland units and their external connectivity with existing cropland. Specifically, the number of shared edges among new cropland units was first calculated to capture their internal clustering and connectivity, and the number of shared edges between new cropland units and existing cropland was then calculated to reflect the extent to which new cropland expands along the margins of existing cropland. These two components were subsequently combined using a weighted summation approach to generate the contiguity index, where higher values indicate a more compact and contiguous configuration. The calculation formula is as follows:

(9) $$\mathrm{SCL}\;=\;\frac{E_{s}\;+\;\lambda \cdot E_{f}}{4N}$$

where *SCL* represents the spatial contiguity of the selected new cropland units; *E_s_* represents the number of shared edges among new cropland units; *E_f_* represents the number of shared edges between new cropland units and existing cropland; *λ* represents the relative importance of the connectivity between new cropland units and existing cropland in the contiguity measure; and 4*N* represents the normalization term, where *N* represents the number of future new cropland raster units.

#### 2.3.3. Optimization Model for New Cropland Allocation

**Figure 3:**
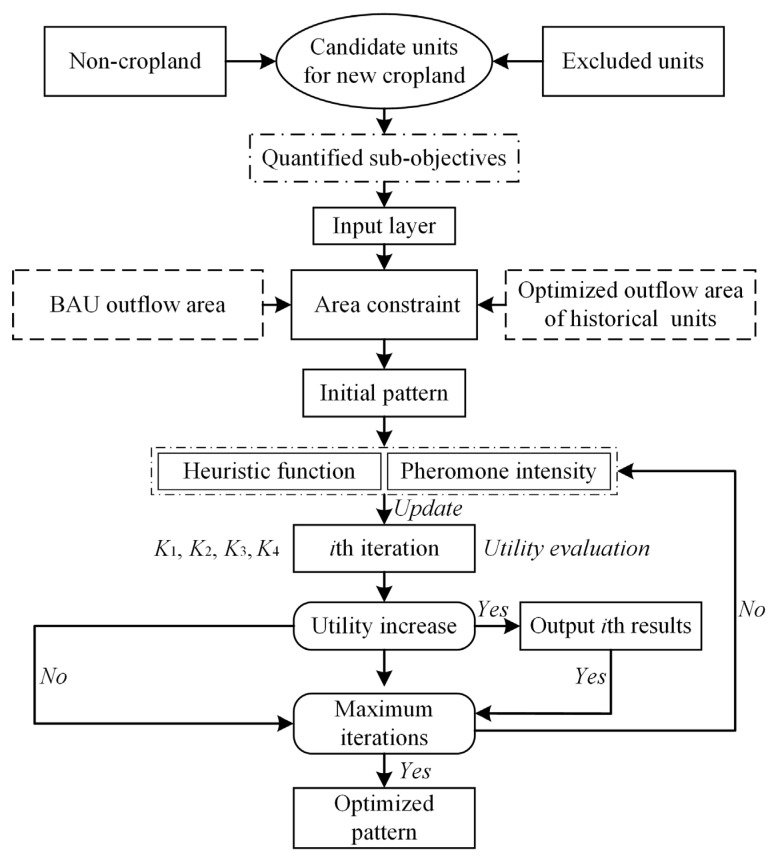
Flowchart of ACO-based spatial optimization for new cropland allocation.

The spatial optimization of new cropland allocation constitutes a complex optimization problem involving trade-offs among multiple objectives, spatial constraints, and combinatorial decision-making. Heuristic algorithms are particularly well suited to this type of large-scale spatial combinatorial optimization problem because they can efficiently explore vast solution spaces and identify high-quality solutions that satisfy prescribed constraints, especially where conventional exact algorithms are difficult to apply directly [14,59]. Ant Colony Optimization (ACO) is a representative heuristic optimization algorithm widely used to address complex combinatorial optimization problems [36,60,61,62]. By exploiting the cooperative search mechanism of artificial ant colonies, ACO provides a suitable approach for optimizing the spatial allocation of new cropland. The ACO-based optimization of future new cropland allocation consists of three main steps (Figure 3). First, subject to constraints on the required area of new cropland and the candidate units available during the planning period, the spatial units eligible for allocation are defined as the search space, thereby establishing the initial optimization state. Second, during the search process, ants continuously adjust the combination of selected units according to pheromone intensity, heuristic information, and utility evaluation, so that allocation results with higher utility are more likely to be retained and reinforced in subsequent iterations, whereas inferior results are progressively eliminated [14,36]. Finally, once the iterative process is completed and the utility becomes stable, the resulting spatial pattern is taken as the optimized allocation of future new cropland.

During the optimization process, the probability that a candidate unit for new cropland is selected by an ant is defined as follows [36]:

(10) $${pro}_{i}^{k}\left(t\right)=\left\{\begin{array}{c} \frac{{\left[T_{i}\left(t\right)\right]}^{\alpha }{\left[\eta_{i}\left(t\right)\right]}^{\beta }}{\sum_{s\in {allowed}_{k}}{\left[T_{s}\left(t\right)\right]}^{\alpha }{\left[\eta_{s}\left(t\right)\right]}^{\beta }},if\;i\in {allowed}_{k}\\ \;0,\;\;\mathrm{otherwise} \end{array}\right.$$

where ${pro}_{i}^{k}(t)$ represents the probability that ant *k* selects unit *i* at time *t*, which is jointly influenced by the pheromone intensity $T_{i}$ and the heuristic function $\eta_{i}$; *α* and *β* represent the relative importance of the pheromone intensity and the heuristic function, respectively; and *allowed_k_* represents the set of candidate units that ant *k* is allowed to visit in the next time.

#### 2.3.4. Scenario Setting and Area Constraint Quantification

To evaluate the optimization effect of planning intervention relative to inertial land use change and to compare the overall differences among alternative allocation pathways in terms of spatial pattern and stable utilization potential of future new cropland, this study considered two scenarios: business-as-usual (BAU) and stable utilization potential enhancement (SUPE). Under the BAU scenario, future new cropland is determined primarily by the historical trajectory of land use change and serves as the baseline for scenario comparison, without incorporating new policy interventions or planning targets. In this scenario, future cropland transitions are governed by historical land use transition rules and key driving factors. By contrast, the SUPE scenario represents an optimized allocation of future new cropland under a fixed area constraint, with explicit emphasis on enhancing stable utilization potential.

With 2035 set as the end year of the planning period, future land use patterns were simulated using the CA–Markov model, and cropland gains identified from the simulation were treated as new cropland under the BAU scenario and used as the baseline for subsequent scenario comparison. The CA–Markov model integrates Cellular Automata (CA) with the Markov model, enabling it to characterize both the quantitative transitions among land use types and the spatial allocation of land use change through neighborhood rules. It has therefore been widely applied in the spatiotemporal simulation and scenario projection of land use change [63,64]. Before the future land use simulation, the 2023 baseline land use map was reconstructed to account for the historical stock of new cropland that had already been withdrawn from cropland use. This step helped to avoid overestimating retained cropland in the baseline map. The withdrawn units from the historical stock of new cropland were then included, together with the projected cropland loss during the planning period, in determining the area constraint for future new cropland allocation. Based on these adjustments, the 2023 land use pattern was updated to generate a reconstructed baseline land use map. On this basis, the land use pattern of the study area by the end of the 2035 planning period was projected in IDRISI Selva 17.0 (Clark Labs, Clark University, Worcester, MA, USA) through four steps, namely land use reclassification, construction of the Markov transition probability matrix, development of the suitability atlas, and model validation. Before the 2035 projection, the simulation performance of the CA–Markov model was evaluated using the Kappa coefficient, overall accuracy, and other accuracy assessment metrics [65,66]. Detailed procedures are provided in Supplementary Text S2. The area constraint for future new cropland was then determined according to the area withdrawn from the historical stock of new cropland and the amount of cropland loss during the planning period.

## 3. Results

### 3.1. Allocation Results for New Cropland Under the BAU Scenario

**Figure 4:**
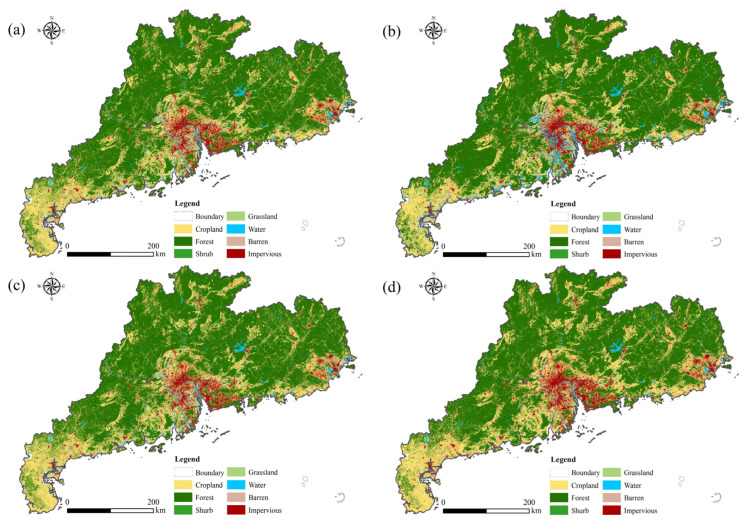
Land use pattern of Guangdong: (**a**) actual 2023 land use pattern; (**b**) simulated 2023 land use pattern; (**c**) adjusted 2023 land use pattern; and (**d**) simulated 2035 land use pattern.

In this study, we used the 2011 land use map as the initial state and parameterized the CA model by incorporating the Markov transition matrix derived from 1999 to 2011 together with a suitability atlas. Under these settings, the number of CA iterations was fixed at 10, and a 5 × 5 filter was applied to regulate neighborhood effects. Based on this configuration, we simulated the land use pattern of the study area for 2023 and subsequently compared it with the observed land use pattern for the same year (Figure 4a,b). Simulation accuracy was evaluated using the Kappa coefficient. The results showed that the Kappa coefficient reached 0.78, while the overall accuracy was greater than 0.85. From the perspective of overall simulation performance, the model produced relatively reliable results. In addition, considering that this study focuses on the identification and allocation of new cropland, the simulated results were further reclassified into cropland and non-cropland, and a confusion matrix was constructed to evaluate cropland simulation accuracy (Table 2). The results showed that the user’s accuracy and producer’s accuracy of cropland were 0.85 and 0.74, respectively, while the overall accuracy was 0.89 and the Kappa coefficient was 0.72. These results indicate that the model captured the spatial distribution of cropland with acceptable accuracy and can support subsequent scenario simulation and optimized allocation analysis of new cropland.

On this basis, the optimized 2023 land use map was adopted as the baseline (Figure 4c), with 2035 defined as the end of the planning period. The land use pattern in 2035 was simulated using the CA–Markov model (Figure 4d). Spatial overlay analysis was then applied to identify both the areas and spatial distribution of cropland inflow and outflow under the BAU scenario. Cropland inflow was regarded as new cropland under the BAU scenario and used as the reference for subsequent scenario comparisons.

**Figure 5:**
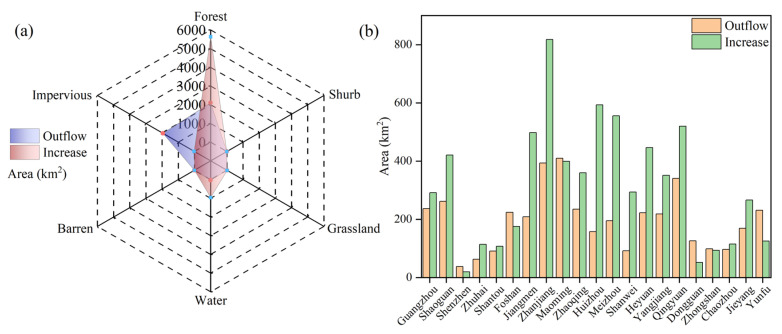
Cropland outflow and increase in area under BAU scenario: (**a**) outflow and increase in area of different land types; and (**b**) outflow and increase in area of different prefecture-level cities.

The results of the BAU scenario indicate that cropland outflow reached 4108.11 km^2^ between 2023 and 2035, mainly converting to forest land and impervious surfaces (Figure 5a). Of this outflow, 51.31% was converted to forest land, while 47.47% was converted to impervious surfaces. During the same period, the area of new cropland amounted to 6614.86 km^2^, primarily derived from forest land and water bodies. Forest-land conversion accounted for 5637.75 km^2^, representing 85.23% of the total inflow, whereas water-body conversion accounted for 959.09 km^2^, representing 14.50% of the total inflow.

At the prefecture level, new cropland was mainly concentrated in Zhanjiang, Huizhou, Meizhou, and Qingyuan (Figure 5b). The total new cropland in these four prefecture-level cities reached 2487.89 km^2^, accounting for 37.60% of the total new cropland area. In addition to these cities, Jiangmen, Zhaoqing, and Guangzhou in the Pearl River Delta also exhibited relatively large increases. By contrast, eastern Guangdong showed a comparatively small expansion, with its new area accounting for only 11.82% of the total increase.

**Figure 6:**
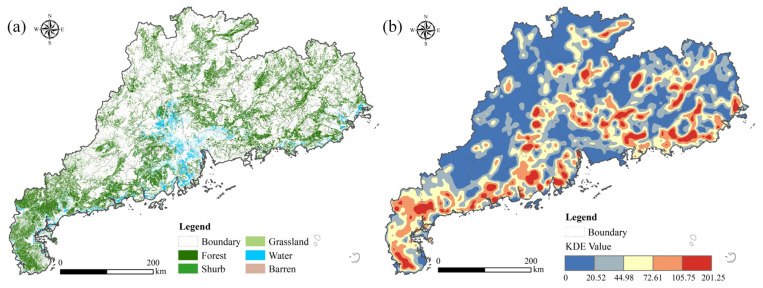
Spatial distribution of new cropland under BAU scenario: (**a**) spatial pattern of different land types converted to cropland; and (**b**) kernel density of new cropland under the BAU scenario.

Spatially, conversions from forest land to cropland were widely distributed, but were mainly concentrated in western Guangdong, northern Guangdong, and some parts of the Pearl River Delta (Figure 6a). In contrast, conversions from water bodies to cropland were primarily distributed in coastal areas and in inland parts of the Pearl River Delta. These areas included Foshan, the southeastern part of Zhaoqing, the northern part of Zhongshan, the southern part of Guangzhou, and parts of Dongguan and Huizhou, where local spatial continuity is relatively strong. The local spatial clustering of new cropland was further examined using Kernel Density Estimation (KDE), with KDE values classified into five levels using the natural breaks method; higher values indicate a stronger tendency toward local concentration of new cropland. The results indicated that high-value areas exhibited an overall dispersed pattern with localized clustering, mostly situated in western Guangdong, the peripheral zones of the Pearl River Delta, and eastern Guangdong (Figure 6b). By comparison, the tendency toward local clustering in northern Guangdong was relatively weak.

### 3.2. Allocation Results for New Cropland Under the SUPE Scenario

#### 3.2.1. Results of Candidate Unit Suitability

(1) Results of stable utilization potential

The hyperparameter search space used for Bayesian optimization is provided in Supplementary Table S2. The final values of the key hyperparameters were max_depth = 20, n_estimators = 1500, learning_rate = 0.03, and min_child_weight = 8, while the detailed settings of the remaining hyperparameters are provided in Supplementary Table S2. For the predictive model constructed using XGBoost in ArcGIS Pro 3.4.3 (Esri, Redlands, CA, USA), the AUC values for the five folds were 96.12%, 96.16%, 96.12%, 96.17%, and 96.17%, respectively. Overall, the model achieved an AUC of 96.15%, an overall accuracy of 90.10%, and an F1 score of 92.42%. These results indicate high predictive accuracy and therefore support the use of the model to estimate the stable utilization potential of candidate units.

The predicted probabilities were normalized using the range normalization method to derive a stable utilization potential index (SUPI), which serves as an indicator of potential stability. The index was classified into three levels using the natural breaks method, namely low stable utilization potential [0–8.63), medium stable utilization potential [8.63–48.63), and high stable utilization potential [48.63–100]. The corresponding area proportions were 71.69%, 17.52%, and 10.79%, respectively. This indicates that candidate units are dominated overall by units with low and medium stable utilization potential, whereas units with high stable utilization potential account for only a small proportion.

**Figure 7:**
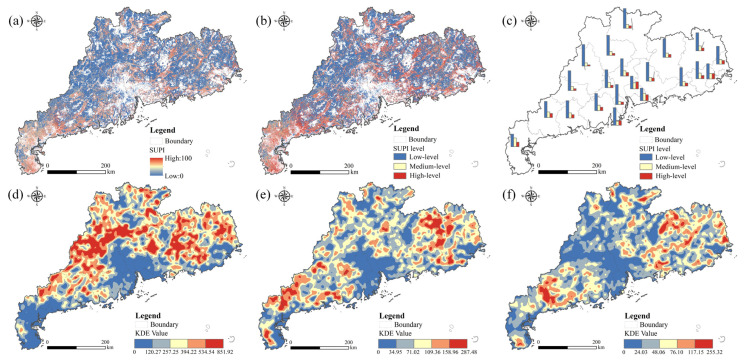
Spatial distribution of stable utilization potential: (**a**) spatial distribution of SUPI; (**b**) spatial distribution of SUPI level; (**c**) proportion of SUPI levels in prefecture-level cities; (**d**) kernel density of low-level SUPI units; (**e**) kernel density of medium-level SUPI units; and (**f**) kernel density of high-level SUPI units.

Spatially (Figure 7), clear differences can be observed among the three levels. Low-level units are widely distributed and form the dominant pattern, and are locally concentrated in northern Guangdong, the peripheral areas of the Pearl River Delta, the western part of Maoming, and both the western and eastern parts of Yangjiang, as well as in contiguous areas of Shanwei and Jieyang. In comparison, medium-level units show a more clustered distribution, and are mainly concentrated in the western and northern parts of Maoming, the southern part of Yangjiang, the Leizhou Peninsula and its northern areas in Zhanjiang, as well as in northeastern and southern Heyuan, the central-western and southwestern parts of Meizhou, and parts of Shanwei and Jieyang. By contrast, high-level units are limited in extent and exhibit clear localized clustering, and are primarily distributed in western Guangdong and in relatively flat areas of northern Guangdong.

Additionally, at the prefecture-level city scale, low-level units account for the largest proportion and therefore dominate the overall pattern. In contrast, the proportion of high-level units ranges from 4.61% to 27.27% across cities. This wide variation indicates strong regional heterogeneity.

(2) Results of production potential

The production potential index (PPI) was classified into three levels using the natural breaks method, namely low production potential [211.25–707.61), medium production potential [707.61–1598.15), and high production potential [1598.15–3934]. The corresponding area proportions are 39.06%, 48.00%, and 12.94%, respectively. As low and medium level units together account for 87.06%, candidate units are dominated by these two categories, while high-level units represent a relatively small proportion.

**Figure 8:**
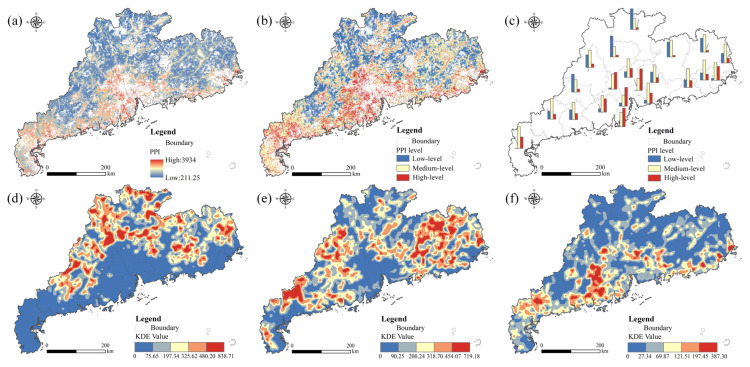
Spatial distribution of production potential: (**a**) spatial distribution of PPI; (**b**) spatial distribution of PPI level; (**c**) proportion of PPI levels in prefecture-level cities; (**d**) kernel density of low-level PPI units; (**e**) kernel density of medium-level PPI units; and (**f**) kernel density of high-level PPI units.

Spatially (Figure 8), low production potential units are mainly distributed in northern Guangdong, Zhaoqing in the Pearl River Delta, and the northeastern part of Maoming in western Guangdong. In comparison, medium-level units are more widely distributed and show localized clustering. They are mostly situated in western Zhanjiang, southwestern Maoming, Zhaoqing, and in Heyuan and Meizhou in northern Guangdong. In contrast, high production potential units are primarily located in lower-latitude areas of the study region. However, in northern Guangdong high-level units are more dispersed, with weaker spatial continuity, and are mainly distributed in the central part of Shaoguan and in relatively flat areas of Heyuan and Meizhou.

At the prefecture-level city scale, the proportion of low production potential is lowest in Dongguan at 0.19% and highest in Qingyuan at 62.51%, indicating substantial variation among cities. For medium production potential, the lowest proportion is observed in Qingyuan at 31.76%, while the highest is found in Zhanjiang at 64.98%. Meanwhile, for high production potential, the lowest proportion occurs in Shaoguan at 5.41%, whereas Dongguan shows the highest proportion at 64.53%.

(3) Results of cost for new cropland

The cost index (CI) of candidate units ranges from 2.11 to 61.01, with a mean value of 23.00. Based on the natural breaks method, the index is classified into three categories, namely low cost [2.11–18.97), medium cost [18.97–27.98), and high cost [27.98–61.01]. The corresponding proportions are 23.42%, 60.44%, and 16.13%, respectively. Overall, the cost for new cropland is relatively low, indicating a certain cost advantage.

**Figure 9:**
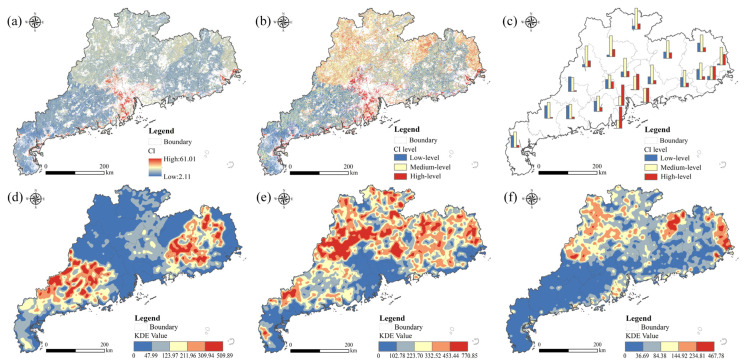
Spatial distribution of CI: (**a**) spatial distribution of CI; (**b**) spatial distribution of CI level; (**c**) proportion of CI levels in prefecture-level cities; (**d**) kernel density of low-level CI units; (**e**) kernel density of medium-level CI units; and (**f**) kernel density of high-level CI units.

From a spatial perspective (Figure 9), low-cost units are mainly distributed in contiguous areas in the western part of the study area, as well as in Heyuan and Meizhou in northern Guangdong and in contiguous areas of eastern Guangdong. In comparison, medium-cost units are mainly located in northern Guangdong. By contrast, high-cost units are concentrated in the central and northern parts of Heyuan, Qingyuan, and northern Zhaoqing in northern Guangdong, as well as in Foshan, Zhongshan, and Zhuhai in the Pearl River Delta, and in parts of central and eastern Chaozhou in eastern Guangdong.

In addition, at the prefecture-level city scale, the proportion of low-cost units is lowest in Zhuhai at 0.09% and highest in Yunfu at 49.63%. For medium-cost units, the lowest proportion is observed in Zhuhai at 27.62%, while the highest occurs in Shaoguan at 70.78%. For high-cost units, the lowest proportion is found in Maoming at 3.34%, whereas Zhuhai shows the highest proportion at 72.29%.

#### 3.2.2. Results of Optimized Allocation

(1) Determination of ACO parameters

To determine the optimal parameter combination, this study first determined the required area of new cropland under the constraint of regional cropland quantity balance policy. This policy aims to maintain the stability of cropland quantity within the region and serves as an important constraint for implementing the requirements of cropland occupation–compensation balance in local land planning and cropland protection management. Specifically, the portion of historical new cropland that had been removed and no longer maintained its cropland use function was 2522.61 km^2^, corresponding to 2,802,900 raster units. The cropland loss during the planning period simulated by the CA–Markov model was 4108.11 km^2^, corresponding to 4,564,563 raster units. Therefore, the total scale that needed to be supplemented through future new cropland allocation was 7,367,463 raster units, corresponding to an area of 6630.72 km^2^. Detailed information on the area composition, raster counts, and area conversions is provided in Supplementary Table S3. For parameters *α*, *β*, and *ρ*, a control variable method was adopted to compare the utility values under different values item by item [36]. The values of *α* and *β* were tested from 0.50 to 5.00 at intervals of 0.50, while *ρ* was tested from 0.05 to 1.00 at intervals of 0.05, with other parameters held constant. A representative area was selected, and the utility value was evaluated under different parameter settings with the objective of maximizing utility. The optimal combination was identified as *α* = 2, *β* = 5, and *ρ* = 0.05. In addition, the utility value stabilized after 600 iterations, and thus the number of iterations *I_t_* was set to 600.

**Figure 10:**
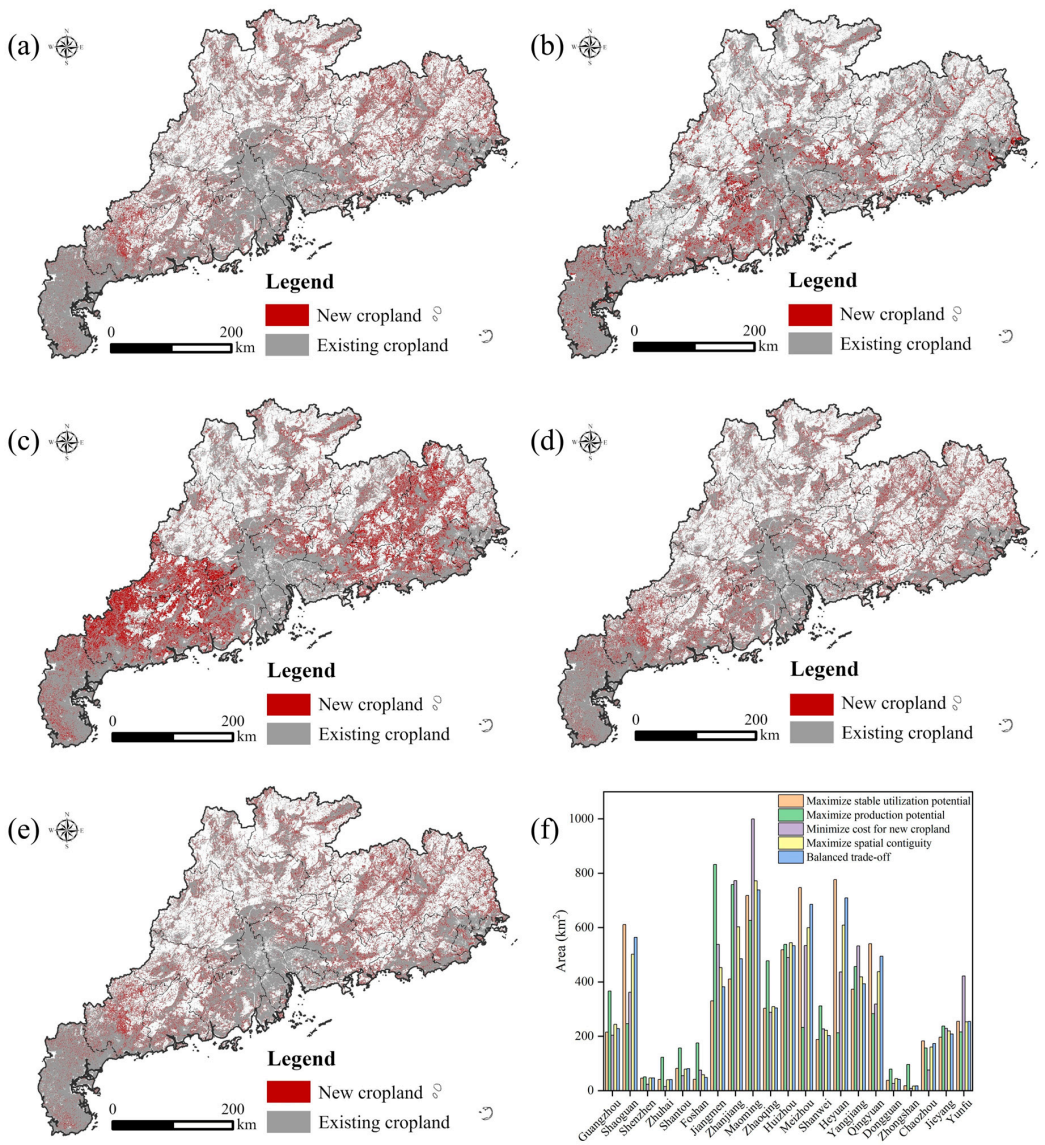
Results under different sub-objective preferences: (**a**) maximize stable utilization potential; (**b**) maximize production potential; (**c**) minimize cost for new cropland; (**d**) maximize spatial contiguity; (**e**) balanced trade-off; and (**f**) Area under different strategies across cities.

Different combinations of sub-objective weights lead to different allocation strategies and reflect varying optimization preferences. Based on this principle, five strategies were designed, including maximizing stable utilization potential, maximizing production potential, minimizing cost for new cropland, maximizing spatial contiguity, and a balanced trade-off strategy. The spatial distribution of new cropland under each scheme is shown in Figure 10. The results indicate clear differences in spatial patterns across strategies, and the new cropland area varies substantially among prefecture-level cities, which highlights the sensitivity of weight settings to outcomes.

A further comparison of the results is presented in Table 3. The strategy maximizing stable utilization potential has an average SUPI of 72.44, 2.13 times that of the maximizing production potential strategy. However, its average production potential index (PPI) is only 60.99% of the latter. In contrast, the production-oriented strategy yields the highest mean PPI of 2249.53, but its SUPI is only 34.05, while its cost is relatively high and its contiguity ratio with existing cropland is relatively low. The cost-minimization strategy achieves the lowest mean cost index (CI), but its SUPI is only 74.09% of that of the stable utilization potential maximization strategy, and its contiguity ratio with existing cropland (CREC) remains limited. The strategies that emphasize spatial contiguity and balanced trade-off both show relatively low cost and high contiguity. However, compared with the balanced trade-off strategy, the maximizing spatial contiguity strategy has a SUPI that is 4.44% lower, while its production potential is 8.74% higher. This reflects clear trade-offs among sub-objectives under different weight combinations. Overall, the balanced strategy performs well across multiple dimensions, achieving a SUPI that reaches 98.61% of that of the stability-oriented scheme while maintaining favorable cost performance and spatial contiguity. Accordingly, the weights of the sub-objectives were set to 0.40, 0.20, 0.20, and 0.20.

(2) Results of optimized allocation

**Figure 11:**
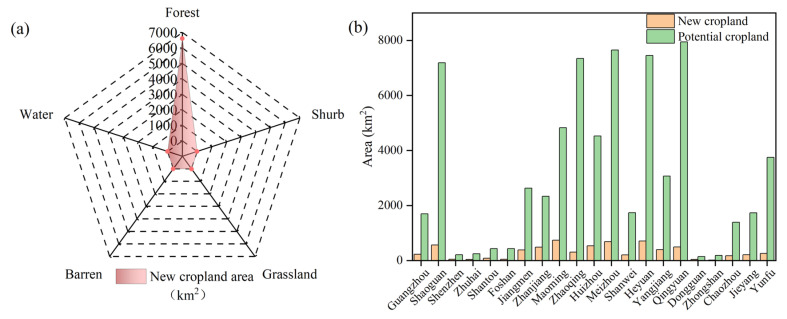
Area of new cropland under the SUPE scenario: (**a**) new cropland area derived from different land types; and (**b**) new cropland area and potential cropland area across prefecture-level cities.

Based on the selected parameter combination, the spatial allocation of new cropland under the SUPE scenario was determined. The results show that conversions from forest land account for 6609.22 km^2^, representing 99.68% of the total, while contributions from other land types account for only 0.32%, as shown in Figure 11a. Compared with the BAU scenario, the proportion of forest land conversion increases markedly, whereas the contribution from water bodies declines substantially. From a regional perspective (Figure 11b), new cropland under the SUPE scenario is mainly concentrated in northern Guangdong, which accounts for 40.84% of the total. The Pearl River Delta, western and eastern Guangdong account for 24.74%, 24.38%, and 10.04%, respectively. When considering the proportion of new cropland to potential cropland, western Guangdong ranks highest at 15.80%, followed by eastern Guangdong at 12.58%, while the Pearl River Delta and northern Guangdong account for 9.42% and 7.97%, respectively. Northern Guangdong boasts the largest area of new cropland. However, it accounts for merely 7.97% of the potential new cropland area, owing mainly to its considerably large base of potential cropland, which makes up 50.78% of the total potential cropland area in the study area.

**Figure 12:**
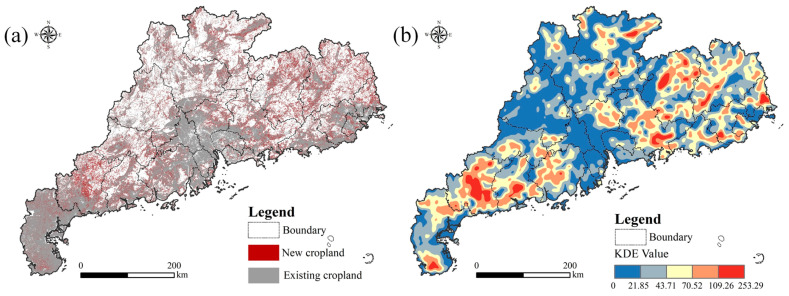
Optimized new cropland allocation under the SUPE scenario: (**a**) spatial distribution of new cropland under the SUPE scenario; and (**b**) kernel density of new cropland under the SUPE scenario.

Spatially, new cropland is generally dispersed, but exhibits pronounced local clustering in certain areas, particularly in central Maoming and the southwestern and central parts of Yangjiang in western Guangdong; central and southwestern Heyuan, as well as the southwestern and northern parts of Meizhou in northern Guangdong; central and eastern Huizhou and eastern Guangzhou in the Pearl River Delta; and eastern Chaozhou together with parts of Jieyang and Shanwei in eastern Guangdong (Figure 12). These areas are characterized by relatively high stable utilization potential and low cost, and thus exhibit higher overall suitability and a greater likelihood of selection. In contrast, new cropland in other areas is more scattered and shows weaker clustering characteristics.

### 3.3. Comparison of Results Under Different Scenarios

#### 3.3.1. Comparison of Comprehensive Utility and Key Indicators

To evaluate the allocation performance of the SUPE and BAU scenarios, a comparative analysis was conducted from the perspectives of comprehensive utility and key indicators (Table 4). Under the given scale constraints and weight settings, the SUPE scenario achieved a comprehensive utility value of 0.46, which was 12.74% higher than that of the BAU scenario. This indicates that, under the given scale constraints and weight settings, the SUPE scenario improves overall utility through optimized spatial allocation of new cropland.

With respect to key indicators, differences are also evident. In terms of regional cropland area gap (RCAG), the required area to be supplemented under the regional cropland quantity balance constraint was 6630.72 km^2^. This target consisted of the removed portion of historical new cropland and the projected cropland loss during the planning period. Under the BAU scenario, new cropland amounts to 6614.86 km^2^, which fails to achieve the quantity balance of regional cropland and results in a gap of 15.86 km^2^, accounting for 0.24% of the required compensation area. Detailed calculation of the regional cropland area gap is provided in Supplementary Table S4. By contrast, the SUPE scenario fully compensates for the cropland loss and achieves quantity balance. The mean SUPI under the SUPE scenario reached 71.43, representing a 34.84% increase compared with the BAU scenario. The SUPE scenario also reduced the mean CI by 10.22% and the mean ecological benefit loss per unit area (EBLA) by 41.36% relative to the BAU scenario, indicating better cost control and lower ecological loss. The neighborhood cropland proportion (NCP) values under the two scenarios were both approximately 0.91, suggesting relatively similar spatial contiguity. However, the mean PPI under the SUPE scenario was 14.45% lower than that under the BAU scenario, reflecting a trade-off between production potential and stable utilization. Despite this decline, the expected mean production benefit (EMPB) increased by 23.97%, indicating that the higher stability of the SUPE scenario improved the stability-weighted production benefit.

On the whole, the SUPE scenario outperforms the BAU scenario in terms of comprehensive utility, stable utilization potential, cost, and expected mean production benefit. Meanwhile, it also incurs a lower mean ecological benefit loss per unit area, demonstrating that it achieves higher overall allocation efficiency under a multi-objective trade-off framework. Although the SUPE scenario does not show an advantage in average production potential, it achieves better outcomes in stability, cost control, and ecological protection.

#### 3.3.2. Spatial Differences Between Scenarios

**Figure 13:**
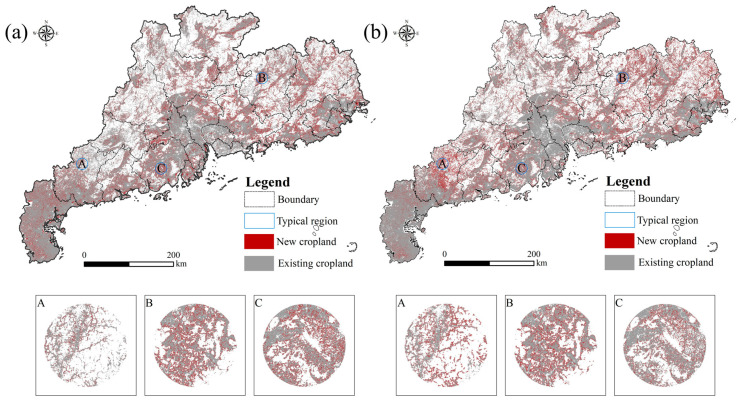
Spatial distribution of new cropland under different scenarios: (**a**) BAU scenario; and (**b**) SUPE scenario.

Spatially, clear differences in the distribution of new cropland were observed between the two scenarios (Figure 13). Under the BAU scenario, new cropland was locally clustered in Zhanjiang, Maoming, and southern Yangjiang in western Guangdong; in peripheral areas of the Pearl River Delta; in contiguous areas of Shanwei and Jieyang in eastern Guangdong; and in eastern Qingyuan as well as central Heyuan and Meizhou in northern Guangdong. Under the SUPE scenario, the pattern of clustering changed markedly. New cropland was mainly located in Maoming in western Guangdong, Huizhou in the Pearl River Delta, and Heyuan and Meizhou in northern Guangdong, while the extent of new cropland in eastern Guangdong declined significantly.

Additionally, some areas showed consistent clustering under both scenarios. These included Zhanjiang and southern Yangjiang in western Guangdong, central and western Jiangmen and central and eastern Huizhou in the Pearl River Delta, Heyuan and central Meizhou in northern Guangdong, as well as central and eastern Chaozhou and contiguous areas of Jieyang and Shanwei in eastern Guangdong.

To further examine spatial differences, three representative regions were selected in Maoming, Zhanjiang, and Heyuan. Region A in western Guangdong showed the largest increase, with new cropland expanding from 15.45 km^2^ under the BAU scenario to 42.35 km^2^ under the SUPE scenario, which was related to its high SUPI, numerous high-stability units, and relatively low development costs. Region B in northern Guangdong showed only a slight increase from 57.81 km^2^ to 62.66 km^2^, as expansion under both scenarios occurred mainly along the margins of existing cropland and maintained strong spatial contiguity. By contrast, Region C in the southern Pearl River Delta decreased from 59.76 km^2^ to 41.66 km^2^ under the SUPE scenario, which was associated with reduced stability related to agricultural structural adjustments and higher ecological compensation costs caused by extensive water bodies.

## 4. Discussion

### 4.1. Practical Applicability of the Proposed Optimization Framework in New Cropland Planning and Management

Future cropland allocation optimization provides an important foundation for achieving high-quality resource utilization in the new era. In this study, we integrated multidimensional assessment with heuristic algorithms to develop an optimization framework for future cropland allocation, centered on enhancing the potential for stable utilization. Under the constraint of maintaining regional cropland quantity balance, this framework enables the spatial optimization of future new cropland and demonstrates practical value for cropland planning and site selection management.

First, it incorporates future stable utilization potential into the cropland allocation process as the core optimization criterion for optimizing future new cropland. Compared with studies that primarily emphasize suitability assessment [67,68,69], this approach can reduce the risk of subsequent unstable use at the allocation stage, thereby helping to improve the long-term feasibility of the allocated cropland. Meanwhile, although a limited number of studies have considered utilization stability, such assessments are typically based on static judgments derived from current conditions [54,70]. In contrast, this study predicts the stable utilization potential of candidate units based on the long-term utilization trajectories of new cropland using a machine learning model. By explicitly accounting for historical patterns of land use change in historically new cropland, this prediction-based approach improves, to some extent, the reliability of stable utilization potential estimates. More importantly, incorporating stable utilization potential as a core optimization objective enhances the scientific rigor of future cropland allocation, while also contributing to more efficient use of cropland development inputs.

Second, with stable utilization potential as its central objective, the framework simultaneously accounts for production potential, cost for new cropland, and spatial contiguity. Compared with single-objective optimization approaches [25,71], this framework jointly considers multiple objectives, thereby enhancing the practical feasibility of the optimization results. In addition, the framework exhibits considerable flexibility. The sensitivity analysis further demonstrates that adjusting the weights assigned to different objectives can generate spatial optimization schemes oriented toward different policy preferences, with marked differences in both the structural composition and spatial layout of new cropland across schemes. This enables planners and land managers to recalibrate objective weights in line with policy priorities while safeguarding the future stable utilization of new cropland, thereby achieving spatial allocation outcomes with differentiated policy orientations.

Third, the framework enables the iterative identification of new cropland units from candidate parcels under a predetermined area constraint. Compared with conventional zoning-based studies or suitability assessment approaches [67,72,73], it moves beyond the limitations of suitability evaluation, which primarily focuses on development feasibility and the identification of relative priorities among land units. Instead, it advances from suitability appraisal to optimization-based allocation, thereby directly supporting the spatial allocation of new cropland and the generation of allocation results, with fewer intermediate screening steps. In addition, the framework incorporates a land use simulation model and determines the required scale of new cropland during the planning period in conjunction with projected cropland loss. This links the estimation of future compensation demand with the spatial allocation of new cropland. Compared with studies that allocate new cropland directly on the basis of static current conditions [13,25], this framework provides a more targeted allocation logic by connecting projected cropland loss with the spatial selection of future new cropland.

### 4.2. Comparison with Other Studies

In terms of the optimization results, although both this study and earlier research [13,17,54] identify a tendency for new cropland to cluster in areas with relatively favorable conditions, the interpretation of this phenomenon differs. Previous studies have generally relied on comprehensive suitability assessments based on current site characteristics, and thus their results essentially capture the development suitability of candidate land parcels. By contrast, this study predicts stable utilization potential from the long-term use outcomes of historical new cropland and further incorporates this indicator, together with spatial contiguity and cost, into the optimization process. Accordingly, the optimization areas identified in this study are not merely places with favorable current conditions, but areas that combine advantageous baseline conditions with a greater probability of long-term stable use and stronger overall comparative advantages for allocation.

Across the alternative allocation scenarios, the BAU scenario tends to place new cropland in areas with higher production potential. However, the candidate units selected under this scenario generally exhibit lower SUPI and relatively higher costs. This indicates that higher production potential does not necessarily coincide with greater stable utilization potential or lower cost, revealing clear trade-offs among the three objectives. The weight sensitivity analysis further confirms these competing relationships. In essence, the spatial optimization of new cropland is a multi-objective decision problem that requires balancing competing objectives, which is broadly consistent with previous studies on cropland allocation optimization [13,74]. In addition, although both scenarios show some capacity to promote spatial contiguity between new cropland and existing cropland, the processes through which such patterns emerge are fundamentally different. The CA–Markov model relies primarily on neighborhood effects and land use transition inertia, which make cropland expansion more likely to occur around existing cropland; in this sense, contiguity under the BAU scenario arises largely as an emergent outcome of spatial proximity [75,76]. By contrast, the ACO algorithm treats contiguity as an explicit optimization objective and actively reinforces the spatial linkage between new cropland and existing cropland during the allocation process. As a result, the contiguity pattern in the optimized scenario is more directly shaped by planning objectives. Moreover, although some heuristic optimization studies have incorporated contiguity into their frameworks, they have often focused mainly on clustering of newly allocated units themselves [14,70], with relatively limited attention to their spatial contiguity with existing cropland. This may lead to new cropland that appears clustered in itself but remains poorly connected to the existing cropland system, thereby undermining the coherence of the overall allocation pattern. Finally, in terms of land use composition, the optimized scenario contains a markedly lower proportion of water units. This is largely because the BAU scenario places greater emphasis on natural endowment conditions when identifying candidate units, and some water units, owing to their relatively favorable resource conditions, exhibit comparatively high production potential and are therefore more likely to be included in the allocation outcome, a pattern that is consistent with previous findings [17,68,77]. In contrast, once stable utilization potential and cost constraints are incorporated into the optimization process, the likelihood of selecting water units declines substantially.

### 4.3. Limitations and Uncertainties

This study develops an optimization framework for allocating future new cropland to enhance stable utilization potential, thereby offering a practical perspective and methodological reference for new cropland allocation; however, the study still has the following limitations and uncertainties.

First, this study takes Guangdong Province as the case study area, and the proposed framework shows some transferability in terms of methodological procedure, providing a reference for the spatial optimization of new cropland allocation in other regions. Specifically, the stable utilization potential prediction framework and the heuristic algorithm-based spatial optimization model have general applicability in their methodological logic. However, given regional differences in natural endowments, agricultural production conditions, socioeconomic contexts, and land management objectives, the predictor system for stable utilization potential, as well as the composition and weight settings of the optimization objective function, should be locally calibrated. For instance, water availability may be a key limiting factor in arid regions of northwestern China, whereas terrain conditions and labor input may play a more prominent role in hilly and mountainous areas. Therefore, the proposed framework should not be directly applied to other regions without regional calibration. Future research could extend this framework to multiple provinces or different regional types by incorporating local policy requirements, resource and environmental conditions, and agricultural production characteristics, thereby further examining its cross-regional applicability and methodological robustness.

Second, this study used 30 m raster cells as the basic spatial unit for land use simulation and new cropland allocation optimization, which may be affected by spatial resolution and scale effects. On the one hand, 30 m raster data may involve mixed-pixel effects, which can influence land use classification results and further affect land use simulation, candidate unit identification, and optimized allocation outcomes. Moreover, the measurement of the stable utilization level of historical new cropland also depends on annual land use classification results; therefore, classification errors may further affect the selection of training samples for the prediction model. On the other hand, spatial adjacency was mainly calculated based on raster neighborhood relationships. Although this approach can reflect the overall adjacency pattern at the provincial scale, it does not fully represent actual connectivity between land parcels and may still be affected by linear features such as roads, rivers, and ditches. Future research could further integrate more detailed land survey data, parcel boundary data, and field verification information to refine the identification of new cropland, the measurement of stable utilization, and the characterization of spatial adjacency, thereby improving the practical applicability of the optimized allocation results.

Third, the prediction, simulation, and optimization processes still involve uncertainties. A machine learning approach was employed to predict the stable utilization potential of candidate units using the observed long-term utilization outcomes of historical new cropland. Although this approach incorporates empirical evidence from historical land use transitions, it is contingent upon the assumption that past land use evolution retains a degree of continuity into the future. Consequently, when major shifts occur in the policy environment, agricultural production and management practices, or climatic conditions, the predicted stable utilization potential may deviate from actual outcomes. In addition, the target scale of new cropland in this study was linked to land use simulation outputs. While this linkage strengthens the temporal relevance of the allocation process, both the target scale and its spatial allocation remain sensitive to the selection of simulation models, parameterization, and scenario assumptions [78]. In particular, the magnitude and spatial configuration of cropland loss during the planning period are uncertain, and such uncertainty may further propagate into the allocation outcomes for new cropland. Although several quantitative checks were conducted, including CA–Markov validation, five-fold cross-validation of the XGBoost model, standard errors for mean indicators, weight sensitivity analysis, and repeated ACO runs with different random seeds, a full end-to-end uncertainty propagation analysis was not performed. Uncertainty may still propagate from land-use classification, CA–Markov simulation, and XGBoost prediction to the ACO-based spatial optimization results. Because ACO is a stochastic heuristic algorithm, the final optimized scenario was represented by the complete allocation result generated under the selected parameter settings and area constraint, rather than by the common overlap of repeated runs. As a supplementary robustness check, the ACO was repeated four times using different random seeds. Of the 7,367,463 raster units selected in each run, 6,276,354 were consistently selected across all four runs, corresponding to an overlap rate of 85.19% and an area of 5648.72 km^2^. This indicates that the core optimized areas were relatively stable, although some variation remained among marginal or substitutable candidate units. Future research could further incorporate dynamic scenarios, such as climate change, policy constraints, and changes in agricultural management, into the prediction of stable utilization potential [17,24,79,80] and land use simulation, and could apply multiple land use simulation models to compare target scales and spatial allocation patterns under alternative scenarios [81]. In addition, Monte Carlo simulations, ensemble land use simulation, and bootstrap-based machine learning prediction could be used to quantify how uncertainties from land use classification, land use simulation, stable utilization prediction, and ACO-based optimization propagate through the full allocation framework, thereby enhancing the dynamic adaptability and robustness of the results.

Fourth, the optimization results may also be affected by weight settings and decision preferences. This study developed a multi-objective optimization framework and determined the new cropland to be allocated over the planning period by primarily accounting for natural conditions, spatial configuration, and overall costs. Five representative weight combinations were tested to compare allocation outcomes under different trade-off strategies. Although this sensitivity analysis helps reveal the influence of weight changes on the optimization results, limited weight combinations cannot fully cover all possible planning objectives and management preferences. In practice, different regions and decision-makers may assign different priorities to stable utilization potential, production potential, cost control, and spatial contiguity. Moreover, the allocation of future new cropland is influenced not only by external spatial conditions but also by the decision preferences and behavioral responses of land operators and project-implementing entities [17,82]. Due to data limitations, stakeholder preferences and behavioral choices were not incorporated into the current framework, which may constrain the practical feasibility of the allocation outcomes. Future research could integrate evidence from field surveys, expert consultations, participatory weighting methods, and agent-based farmer behavior models into the optimization framework, thereby improving the practical feasibility of the optimized allocation outcomes.

### 4.4. Policy Implications

Based on the key findings of this study, the following policy recommendations are proposed to support the optimized allocation of future new cropland and strengthen regional food security.

(1) Stable utilization potential should be incorporated into future new cropland site selection

Future new cropland site selection should not rely only on natural endowment or production suitability. Stable utilization potential should be incorporated as a key criterion in the screening of candidate units. Areas with higher stable utilization potential, lower risk of subsequent unstable use, acceptable development cost, and reasonable adjacency to existing cropland should be prioritized. By contrast, areas with excessive development costs, strong ecological constraints, or high risks of future unstable use should be excluded where necessary. This can help reduce the risk that newly allocated cropland becomes unstable after development.

(2) Future new cropland allocation should balance multiple objectives

The optimization results show that different objective preferences can lead to substantial differences in the spatial distribution and regional scale of new cropland. A single-objective allocation strategy may improve one target but weaken performance in other dimensions, such as cost control, ecological loss, or spatial contiguity. Therefore, future new cropland allocation should jointly consider stable utilization potential, production potential, development cost, ecological impact, and spatial contiguity. In practice, objective weights should be adjusted according to regional planning priorities and tested through sensitivity analysis to avoid allocation outcomes dominated by a single target.

(3) Post-development management and monitoring mechanism should be strengthened

The establishment of new cropland does not necessarily guarantee sustained and stable use. After development, management authorities should strengthen the maintenance of cropland infrastructure, soil fertility improvement, and cultivation-condition enhancement. A parcel-level monitoring mechanism should also be established to dynamically track the annual utilization status of new cropland. Parcels that fail to meet cultivation requirements or show abnormal use, such as abandonment or conversion to non-cropland uses, should be subject to timely warning, rectification, and classified management.

## 5. Conclusions

The stable utilization of new cropland is fundamental to safeguarding regional food security and achieving the policy targets of cropland protection. To enhance the stable utilization potential of future new cropland, this study incorporated land use transition patterns of historical new cropland units, employed a machine learning model to predict stable utilization potential, and integrated the prediction results with ant colony optimization to develop an optimization framework. This framework focuses on stable utilization potential while also taking into account key objectives such as production potential, costs, and spatial contiguity. It has been empirically validated in Guangdong Province, China, and provides a reference for the optimized allocation of future new cropland. The results indicated that the proposed framework can effectively enhance the stable utilization potential of new cropland. Under the specified objective-weighting scheme and compared with the BAU scenario, the optimized scenario achieved regional cropland quantity balance. It also increased the average stable utilization potential of new cropland and overall utility by 34.84% and 12.74%, respectively, while reducing the mean cost and mean ecological benefit loss per unit area by 10.22% and 41.36%, respectively. Optimized new cropland was mainly concentrated in areas with higher stable utilization potential and lower costs, which also exhibited stronger contiguity with existing cropland and more favorable resource endowments for agricultural production. Based on these findings, we recommend that management authorities should strengthen the stable-utilization orientation in the site selection and quality assessment of new cropland, while also improving post-development management and monitoring mechanisms to support the sustained use of new cropland.

## Figures and Tables

**Table 1:** Data sources and description.

Category	Data	Resolution	Data Sources
Vector data	Administrative boundary data	Shapefile	Department of Natural Resources of Guangdong Province
	The Third National Land Survey data of Guangdong Province	Shapefile	Department of Natural Resources of Guangdong Province
	Ecological redline	Shapefile	Department of Natural Resources of Guangdong Province
Raster data	DEM	30 m	https://www.gscloud.cn/ (accessed on 26 April 2025)
	Soil data	1 km	http://soil.geodata.cn/ (accessed on 26 April 2025)
	CLCD	30 m	https://doi.org/10.5281/zenodo.4417810 (accessed on 22 April 2025)
	CNLUCC	30 m	https://www.resdc.cn/ (accessed on 22 April 2025)
	NTL	1 km	https://doi.org/10.7910/DVN/GIYGJU (accessed on 26 April 2025)
	Precipitation data	1 km	https://data.tpdc.ac.cn/ (accessed on 26 April 2025)
	Temperature data	1 km	https://data.tpdc.ac.cn/ (accessed on 26 April 2025)
	Population data	100 m	https://www.worldpop.org; https://hub.worldpop.org/doi/10.5258/SOTON/WP00839 (accessed on 8 May 2025)

**Table 2:** Confusion matrix and accuracy assessment for cropland simulation.

Types		Observed Cell Number			User’s Accuracy
		Cropland	Non-Cropland	Total	
Simulated cell number	Cropland	38,999,359	7,086,252	46,085,611	0.85
	Non-cropland	13,858,527	137,145,217	151,003,744	0.91
	Total	52,857,886	144,231,469	197,089,355	
Producer’s Accuracy		0.74	0.95		
Overall Accuracy		0.89			
Kappa		0.72			

**Table 3:** Results under different weighting strategies.

Strategies	Weight Combination (*w*_1_, *w*_2_, *w*_3_, *w*_4_)	SUPI (Mean ± SE)	PPI (Mean ± SE)	CI (Mean ± SE)	CREC
Maximize stable utilization potential	0.70, 0.10, 0.10, 0.10	72.44 ± 4.96 × 10^−3^	1371.96 ± 0.24	21.11 ± 1.52 × 10^−3^	0.29
Maximize production potential	0.10, 0.70, 0.10, 0.10	34.05 ± 0.01	2249.53 ± 0.16	25.02 ± 3.41 × 10^−3^	0.20
Minimize cost for new cropland	0.10, 0.10, 0.70, 0.10	53.67 ± 0.01	1565.55 ± 0.24	18.64 ± 1.19 × 10^−3^	0.26
Maximize spatial contiguity	0.10, 0.10, 0.10, 0.70	68.26 ± 0.01	1590.94 ± 0.24	20.48 ± 1.41 × 10^−3^	0.30
Balanced trade-off	0.40, 0.20, 0.20, 0.20	71.43 ± 0.01	1463.09 ± 0.24	20.79 ± 1.46 × 10^−3^	0.30

**Table 4:** Comparative performance of the BAU and SUPE scenarios.

Comparative Indicators	Scenarios	
	BAU	SUPE
Comprehensive utility	0.41	0.46
RCAG	15.86	0.00
SUPI (Mean ± SE)	52.98 ± 0.01	71.43 ± 0.01
PPI (Mean ± SE)	1710.25 ± 0.27	1463.09 ± 0.24
CI (Mean ± SE)	23.15 ± 3.18 × 10^−3^	20.79 ± 1.46 × 10^−3^
NCP	0.91	0.91
EMPB (Mean ± SE)	0.19 ± 5.94 × 10^−5^	0.24 ± 5.13 × 10^−5^
EBLA (Mean ± SE)	35.67 ± 0.01	20.92 ± 2.06 × 10^−4^

## Data Availability

The original contributions presented in the study are included in the article and Supplementary Materials, further inquiries can be directed to the corresponding author.

## Supplementary Materials

The following supporting information can be downloaded at: https://www.mdpi.com/article/10.3390/foods15111845/s1, Table S1: Predictor variables system and feature importance ranking; Table S2: Hyperparameter search space and optimal values of the XGBoost model; Table S3: Composition of the required area for new cropland allocation; Table S4: Regional cropland area gap; Text S1: Pseudocode of the ACO-based spatial allocation algorithm; Text S2: The detailed procedures of the CA-Markov model. References [17,83,84,85,86] are cited in the Supplementary Materials.
