# Supplementary material for: Optimization of New Cropland Allocation to Enhance Stable Utilization Potential: A Case Study of Guangdong Province, China
Source: Foods. 2026 May 23;15(11):1845. doi: 10.3390/foods15111845 (PMC13257041; doi:10.3390/foods15111845)
Supplement: Supplementary file 1 [file foods-15-01845-s001.zip › foods-4271183-Supplementary materials.pdf]

Supplementary materials

S*1: *Supplementary Tables

Supplementary Table S1. Predictor variables system and feature importance ranking.

Factor layer	Indicator layer	Feature importance ranking
Natural endowment	Elevation	14
	Slope	24
	Terrain relief	12
	Soil depth	16
	Soil organic carbon content	20
	pH deviation	21
	Soil texture	17
	Temperature	7
	Precipitation	15
Agricultural production and management	Distance to water source	6
	Cultivation distance	9
	Ditch accessibility	8
	Road accessibility	19
	Elevation difference	10
	Aggregation area	3
	Shape index	5
Socioeconomic conditions	Distance to towns	18
	Nighttime light index	13
	Total population	23
	Labor force ratio	22
	Aging population ratio	11
Neighborhood land use structure	Proportion of cropland in the neighborhood	1
	Proportion of built-up land in the neighborhood	4
	Proportion of ecological land in the neighborhood	2

Note: Feature importance ranking was derived from the XGBoost gain metric and averaged across the five-fold cross-validation procedure; smaller rank values indicate higher importance.

Supplementary Table S2. Hyperparameter search space and optimal values of the XGBoost model.

Hyperparameter	Search space	Optimal value
max_depth	[4, 8, 12, 16, 20, 25, 30, 35, 50]	20
n_estimators	[500, 1000, 1500, 2000, 2500, 3000, 4000]	1500
learning_rate	[0.01, 0.03, 0.05, 0.08, 0.1, 0.2, 0.5]	0.03
min_child_weight	[1, 3, 5, 8, 12, 16]	8
subsample	[0.2, 0.4, 0.6, 0.8, 1.0]	1
colsample_bytree	[0.2, 0.4, 0.6, 0.8, 1.0]	0.6
reg_lambda	[0, 1, 3, 5, 8]	5
reg_alpha	[0, 0.1, 0.5, 1.0, 2.0]	2
gamma	[0, 0.1, 0.5, 1, 2, 3]	0

Supplementary Table S3. Composition of the required area for new cropland allocation

Component	Raw raster count	Area per raster unit (m^2)	Converted area (km^2)	Role / interpretation	Calculation
Historical new cropland no longer retaining cropland use	2,802,900	900	2,522.61	Historical new cropland included in the area constraint because it no longer retained cropland use	2,802,900 × 900 / 1,000,000
Projected cropland loss during the planning period	4,564,563	900	4,108.11	Projected future cropland loss simulated by the CA-Markov model	4,564,563 × 900 / 1,000,000
Required future new cropland allocation scale	7,367,463	900	6,630.72	Target allocation scale in the ACO model	(2,802,900 + 4,564,563) × 900 / 1,000,000

Supplementary Table S4. Regional cropland area gap

Component	Raster units	Area per raster unit (m^2)	Area (km^2)	Percentage	Clarification
Required future new cropland allocation scale	7,367,463	900	6,630.72		Total compensation requirement, including historical new cropland no longer retaining cropland use and projected future cropland loss
Simulated new cropland under the BAU scenario	7,349,844	900	6,614.86		BAU simulated new cropland area used for scenario comparison
Regional cropland area gap under the BAU scenario	17,619	900	15.86	0.24%	Difference between the required allocation scale and BAU simulated new cropland area

S2*: *Supplementary Texts

Supplementary Text S1: Pseudocode of the ACO-based spatial allocation algorithm

Initialize the candidate unit set C and set the corresponding parameters α, β, ρ, the required number of selected units K, and the maximum number of iterations I_t.

Define the objective function U(S) for evaluating each allocation solution.

Initialize the pheromone intensity T_i of each candidate unit.

Set the global-best solution S_{best} as empty and initialize its objective function value U_{best} as a very small value.

Initialize the iteration counter $t = 1$.

%Main Loop

While ($t \leq I_t$)

Set the iteration-best solution S_{iter} as empty and initialize its objective function value as a very small value.

For (each ant k) do

Initialize the allocation solution S_k as empty.

Set $allowed_k$ as the set of candidate units that ant k is allowed to select.

While (the number of selected units in S_k is less than K) do

Calculate the selection probability $pro_i^k(t)$ for each candidate unit i in $allowed_k$ according to the pheromone intensity T_i and the heuristic function η_i.

Select one candidate unit according to $pro_i^k(t)$.

Add the selected unit to S_k and remove it from $allowed_k$.

End while

Calculate the objective function value $U(S_k)$.

If (S_k is better than the current iteration-best solution) then

Replace the current iteration-best solution S_{iter} with S_k.

End if

End for

Update the pheromone intensity of all candidate units through pheromone evaporation.

Reinforce the pheromone intensity of the units included in S_{iter}.

If (S_{iter} is better than the global-best solution) then

Replace the global-best solution S_{best} with S_{iter}, and update U_{best}.

End if

Update the iteration counter: $t = t + 1$.

End while

Return S_{best} and its objective function value U_{best}.

Supplementary Text S2: The detailed procedures of the CA-Markov model

(1) Land use reclassification

To satisfy the CA-Markov model requirement for a unified classification system of discrete land use types, the land use data for each period were reclassified prior to model implementation. This process mainly involved three steps. First, the coordinate system, spatial resolution, and spatial extent of land use data from different periods were standardized. Second, according to the research objective, the original land use classes were regrouped into seven categories: cropland, forest, shrub, grassland, water, barren, and impervious surfaces. Third, each reclassified land use type was assigned a unique integer code to ensure that both the semantic meaning and coding of land use categories remained consistent across different periods, thereby providing a reliable basis for scenario simulation.

(2) Construction of the Markov transition probability matrix

Based on the reclassified land use data, the Markov module in IDRISI Selva was used to calculate the land use transition probability matrices for 1999–2011 and 2011–2023 on the basis of land use and land cover data for 1999, 2011, and 2023. Among these, the transition probability matrix for 1999–2011 was used to predict the quantitative structure of land use types in 2023, whereas the matrix for 2011–2023 was used to predict the quantity of each land use type in 2035. In addition, based on the optimization results for the use reallocation of historical stock new cropland, this study reconstructed the 2011–2023 transition probability matrix and, on this basis, determined the quantity of each land use type at the end of the planning period.

(3) Development of the suitability atlas

In this study, the suitability atlas was generated using the Multi-Criteria Evaluation (MCE) method, which mainly involved three steps. First, the restriction factors and continuous constraint factors for each land use type were identified. For cropland, these restriction factors included ecological redline areas, areas with slopes greater than 25°, urban, rural, industrial and mining land areas, natural water bodies, impervious surface, and areas within historical stock new cropland that had been designated as non-cropland. Continuous constraint factors were continuous variables used to reflect the degree to which different factors influenced land use types. In this study, ten continuous constraint factors were selected from both natural and socioeconomic dimensions[17,

83-85]. The natural factors mainly included elevation, slope, mean annual precipitation, mean annual temperature, and distance to the water, whereas the socioeconomic factors mainly included population, nighttime light index, distance to towns, distance to roads, and distance to rural settlements. Second, the Analytic Hierarchy Process was used to assign weights to each factor for each land use type so as to measure the relative importance of different factors for different land use transitions, and the continuous constraint factors were standardized using the FUZZY module. Finally, the suitability atlas for each land use type was produced in the MCE module based on the suitability factors and restriction factors.

(4) Model validation

Finally, simulation and validation were conducted. Using the 2011 land use map as the base, the land use pattern in 2023 was simulated, and model accuracy was validated using the Kappa coefficient. If the validation was satisfactory, the land use pattern in 2035 was then further simulated. A Kappa coefficient between 0.75 and 1.00 indicates a high degree of agreement between the simulated and observed results; a value between 0.50 and 0.75 indicates moderate agreement; and a value below 0.50 indicates little or no agreement[86].
